# Understanding the impact of the gut microbiome on opioid use disorder: Pathways, mechanisms, and treatment insights

**DOI:** 10.1111/1751-7915.70030

**Published:** 2024-10-10

**Authors:** Negin Kazemian, Sepideh Pakpour

**Affiliations:** ^1^ School of Engineering University of British Columbia Kelowna British Columbia Canada

## Abstract

The widespread use of opioids for chronic pain management not only poses a significant public health issue but also contributes to the risk of tolerance, dependence, and addiction, leading to opioid use disorder (OUD), which affects millions globally each year. Recent research has highlighted a potential bidirectional relationship between the gut microbiome and OUD. This emerging perspective is critical, especially as the opioid epidemic intensifies, emphasizing the need to investigate how OUD may alter gut microbiome dynamics and vice versa. Understanding these interactions could reveal new insights into the mechanisms of addiction and tolerance, as well as provide novel approaches for managing and potentially mitigating OUD impacts. This comprehensive review explores the intricate bidirectional link through the gut–brain axis, focusing on how opiates influence microbial composition, functional changes, and gut mucosal integrity. By synthesizing current findings, the review aims to inspire new strategies to combat the opioid crisis and leverage microbiome‐centred interventions for preventing and treating OUD.

## Graphical abstract

The gut environment is shaped by various interacting elements including the gut microbiota, metabolites, and mucus, all of which bidirectionally communicate with the brain via the gut–brain axis. These elements collectively influence the overall state and function of the gut and play significant roles in opioid use disorder (OUD).

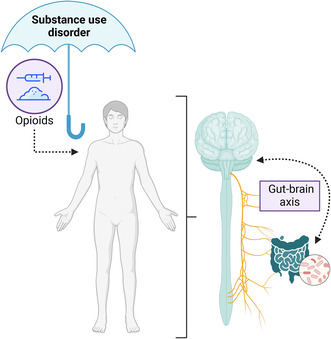

## INTRODUCTION

**FIGURE 1:**
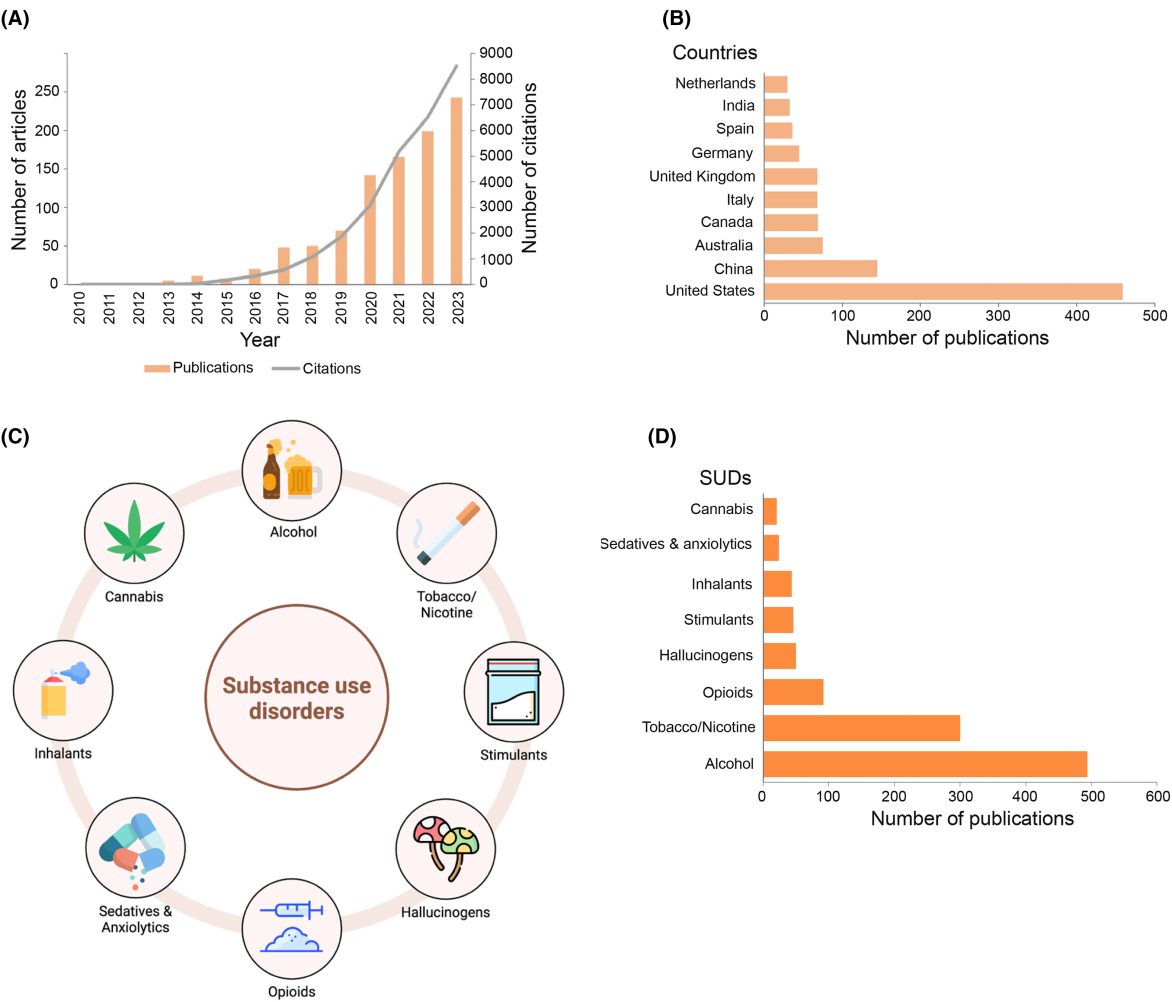
Overview of research trends and contributions in substance use disorder (SUDs) and the gut microbiome. Trends in the number of articles listed in the Scopus database from 2010 to 2023 and corresponding citations for research on SUDs and the gut microbiome (A) The top 10 countries contributing research articles on SUDs and the gut microbiome (B) Classification of substances under SUDs (C). Distribution of publications among different classes of SUDs and the gut microbiome (D). Data for A, B, and D were retrieved from the Scopus database (https://www.scopus.com) from 2010 to 2023. Figure C was created with BiorRender.com.

Substance use disorders (SUDs) constitute a multifaceted and pervasive global health challenge with 46.3 million people aged 12 or older having SUDs in the United States alone in 2021 (Substance Abuse and Mental Health Services Administration, 2022). Characterized by the compulsive and harmful consumption of substances, SUD extends its reach across diverse demographics and socioeconomic strata, exerting profound impacts on individual well‐being, social cohesion, and economic stability (Fonseca et al., 2021; McCabe et al., 2007). Recent research has illuminated the pivotal role of the gut microbiome in the context of substance abuse, offering novel insights into addiction aetiology and potential therapeutic avenues (Russell et al., 2021). Specifically, within the last decade, SUDs and their relationship with the gut microbiome have garnered increasing attention, evidenced by the surge in the publication of research articles and citations addressing this multifaceted relationship (Figure 1A). Based on the data retrieved from the Scopus database, from 2010 to 2023, the United States, China, and Australia were the top 3 contributors to research articles regarding SUDs and gut microbiome, followed by Canada, Italy, United Kingdom, Germany, Spain, India, and Netherlands, reflecting the global interest and collaboration in this critical area of study (Figure 1B).

Substances encompassed within SUDs range from alcohol, tobacco, cannabis, and inhalants, to a myriad of illicit drugs such as stimulants, hallucinogens, sedatives and anxiolytics, and opioids (Figure 1C). Among the various forms of SUDS, opioid use disorders (OUDs) have emerged as a focal point of concern, exacerbated by the pervasive over‐prescription for pain management (The Lancet Regional Health‐Americas, 2023). The opioid class encompasses various types of drugs including natural opiates such as morphine and codeine, semi‐synthetic opioids such as heroin, hydromorphone, oxycodone and hydrocodone, as well as fully synthetic opioids such as fentanyl, and tramadol (Shafi et al., 2022). Fentanyl and its analogs, in particular, have garnered significant attention due to their potency and involvement in numerous overdose deaths worldwide, with potency levels exceeding 50 times that of heroin and 100 times of morphine (Centers for Disease Control and Prevention. Fentanyl Facts, 2023; The Lancet Regional Health‐Americas, 2023). The proliferations of opioids, including those prescribed for pain management, has catalysed an opioid epidemic, leading to millions grappling with OUD as a consequence of over‐prescription and misuse (Judd et al., 2023; The Lancet Regional Health‐Americas, 2023). Morphine, for example, is the gold standard for pain management. However, its widespread usage is curtailed by significant adverse effects, including addiction, the development of analgesic tolerance, immunosuppression, and gastrointestinal (GI) symptoms (Kosciuczuk et al., 2020; Wang et al., 2018). Opioid use has been shown to disrupt gut homeostasis, leading to microbial dysbiosis and functional consequences, and the interplay between opioid use and chronic conditions such as cirrhosis and inflammatory bowel disease (IBD) underscores the complex landscape of opioid‐related morbidity and mortality (Acharya et al., 2017; Niccum et al., 2020; Targownik et al., 2014). In addition, the studies have revealed mechanisms by which the microbiome affects its host's brain, behaviour and well‐being, and that dysbiosis – persistent microbiome imbalance – is associated with the onset and progress of various chronic diseases, including addictive behaviours (Fu et al., 2021; Luo et al., 2023). However, surprisingly, the number of publications exploring the relationship between OUD and the gut microbiome from 2010 to 2023 has remained limited, totalling only 92 publications listed in the Scopus database, in stark contrast to the extensive research attention directed towards tobacco (300 publications) and alcohol (494 publications) in conjunction with the gut microbiome (Figure 1D). Thus, despite advancements in understanding the bidirectional relationship between the gut microbiome and OUD, significant gaps persist in elucidating the underlying mechanisms shaping this intricate interplay. Therefore, this review aims to shed light on the knowledge of the interconnection between OUD and the gut microbiome, delineating its implications for preventative strategies and treatment interventions.

## OUD AND GUT MICROBIOME ALTERATIONS

OUD exerts a significant influence on the gut ecosystem, manifesting in a myriad of GI symptoms including constipation, bloating, nausea, and vomiting, indicative of its profound effects on the functions of the GI tract, such as digestion, absorption, secretion, motility, immune surveillance, and tolerance (Lang‐Illievich & Bornemann‐Cimenti, 2019; Wang & Roy, 2017). Opioid treatment is known to attenuate intestinal motility by inhibiting coordinated myenteric activity, thus delaying transit time and potentially increasing the risk for bacterial translocation in humans (Galligan & Sternini, 2017; Leppert, 2012; Poulsen et al., 2016). Moreover, chronic opioid use has been associated with notable alterations in gut microbial diversity and abundance, evidenced by both animal (Ghosh et al., 2023; Johnson et al., 2022; Kang et al., 2017; Lee et al., 2018; Wang et al., 2018) and human studies (Acharya et al., 2017; Cruz‐Lebrón et al., 2021; Gicquelais et al., 2020). These microbial changes are thought to be both a consequence of opioid‐induced alterations in gut physiology and a potential contributing factor to the GI symptoms observed in opioid users. Opioid‐induced reductions in gut motility and changes in gut secretions create an environment that disrupts the balance of gut microbiota. For example, decreased transit time can lead to increased microbial overgrowth and dysbiosis, while alterations in gut secretions may affect microbial nutrient availability and growth (Grace et al., 2013). Conversely, microbial dysbiosis itself can exacerbate GI symptoms by affecting gut inflammation, barrier integrity, and the production of metabolites that influence GI function (Singh et al., 2021). Therefore, the relationship between opioid use, gut microbiome alterations, and GI symptoms is complex and bidirectional.

In the following sections, we delve into the specific impacts of various opioids on the gut microbiome, drawing from a range of studies to highlight differences and commonalities across different opioid treatments.

### Morphine

The majority of studies on opioid‐induced gut microbiota alterations focus on animals exposed to morphine, a widely used opioid analgesic. Studies have consistently demonstrated that morphine administration significantly disrupts the gut microbial ecosystem, leading to a marked decrease in overall microbial diversity (Ghosh et al., 2023; Kang et al., 2017; Wang et al., 2018). For example, a study by Wang et al. (2018) observed a significant reduction in gut microbial diversity with a 13% decrease in the chao1 index in morphine‐treated mice compared to the placebo group. Similarly, there was a slight but significant reduction in the total bacterial load in faecal samples of morphine‐treated mice, determined by 16S rRNA gene copy numbers (Kang et al., 2017). Further supporting these findings, Ghosh et al. (2023) showed significant differences in the Shannon index, with the morphine‐treated group exhibiting less bacterial diversity compared to the placebo group. Complementing these murine studies, decreased α‐diversity was also reported in morphine‐treated rhesus macaques, suggesting that the disruptive effects of morphine on gut microbiota diversity are consistent across different animal models (Johnson et al., 2022). However, contradictory findings have been reported, with some studies finding no impact on bacterial α‐diversity in morphine‐treated mice (Banerjee et al., 2016; Lee et al., 2018) and rats (Zhang et al., 2020). These discrepancies highlight the complexity of morphine's effects on gut microbiota and suggest that factors such as dosage, duration, and experimental conditions may influence outcomes.

Building on the observed alterations in overall microbial diversity, studies have also documented significant changes in the composition of the gut microbiota (beta diversity) in response to morphine exposure. These compositional shifts are characterized by notable increases in certain bacterial taxa, including pathogenic genera, alongside reductions in beneficial commensal populations. For instance, the studies have documented an overrepresentation of the Firmicutes phylum and a decline in the abundance of Bacteroidetes and Actinobacteria in morphine‐treated mice, resulting in a reduced Bacteroidetes/Firmicutes ratio (Banerjee et al., 2016; Ghosh et al., 2023), which is often associated with an increased level of inflammation (Stojanov et al., 2020). At the family level, the studies have shown an elevated abundance of Enterococcaceae, Staphylococcaceae, Peptostreptococcaceae, Streptococcaceae, Erysipelotrichaceae, Pseudomonaceae, Akkermansiaceae, Coriobacteriaceae, Neisseriaceae, and Bacillaceae in the morphine‐treated group (Banerjee et al., 2016; Ghosh et al., 2023). Conversely, a reduction in the abundance of Lactobacillaceae, Lachnospiraceae, Muribaculaceae, Ruminococcaceae, Burkholderiaceae, Eggerthellaceae, and Peptococcaceae was observed in these mice (Ghosh et al., 2023). Morphine treatment in mice has been associated with an increased prevalence of several bacterial genera, including *Clostridium*, *Enterococcus*, *Sutterella*, *Fusobacterium*, *Flavobacterium*, *Parabacteroides*, *Staphylococcus*, and *Ruminococcus* (Lee et al., 2018; Meng et al., 2015; Wang et al., 2018). On the other hand, there was a significant reduction in *Lactobacillus*, a genus known for its role in preventing inflammation and maintaining barrier function, in the morphine‐treated group (Banerjee et al., 2016; Ghosh et al., 2023; Lee et al., 2018; Meng et al., 2015). Using metagenomic sequencing in an in vivo mouse model, Kolli et al. (2023) revealed that morphine treatment resulted in significant expansion of *Parasuterella excrementihominis*, *Burkholderiales bacterium 1_1_47*, *Enterococcus faecalis*, *Enterorhabdus caecimuris* and depletion of *Lactobacillus johnsonii*.

In parallel to these findings, human studies have observed similar trends. In cirrhotic in‐patients using various opioids, including morphine, there was a notable decrease in the abundance of Clostridiales XIV, Ruminococcaceae, and Bacteroidaceae (Acharya et al., 2017). Morphine‐induced alterations in the gut microbiome can exacerbate gastrointestinal symptoms such as constipation and abdominal discomfort, highlighting the broader implications of opioid use on gut health (Lang‐Illievich & Bornemann‐Cimenti, 2019). Understanding these microbial changes is crucial for managing the side effects of morphine and developing strategies to mitigate its impact on gut microbiota and overall health.

### Heroin

In a study conducted by Gicquelais et al. (2020), the impact of opioid agonists (heroin or prescription opioids) and antagonists on gut microbiota was explored, revealing a reduced α‐diversity in the opioid agonist group. While some studies reported a significant reduction in α‐diversity, indicating a disrupted and less diverse gut ecosystem (Gicquelais et al., 2020; Yang, Xiong, et al., 2021a), others found no change in α‐diversity associated with heroin use (Greenberg et al., 2024). Despite these differences in α‐diversity outcomes, consistent alterations in specific bacterial groups have been observed. For instance, Gicquelais et al. (2020) identified increased abundances of Enterobacteriaceae, *Lactobacillus*, *Clostridium* cluster XIVa, *Faecalicoccus*, *Anaerostipes*, and *Streptococcus*, coupled with a reduction in Firmicutes, *Bilophila*, and *Roseburia* among heroin or prescription opioid users. Greenberg et al. (2024) found an increase in *Bacteroides* and Muribaculaceae, alongside a decrease in Rikenellaceae, *Alistipes*, and Lachnospiraceae. Moreover, in a mouse model with heroin dependence, Yang, Xiong, et al. (2021a) found higher levels of *Bifidobacterium* and *Sutterella*, with a concurrent decrease in *Akkermansia* at the genus level, supporting the notion that heroin use can significantly alter gut microbial composition. The disruption of the gut microbiome induced by heroin underscores the importance of understanding its impact on gut health and its potential implications for opioid‐related comorbidities and overall health.

### Hydromorphone

Hydromorphone, a potent opioid analgesic, has been shown to impact the gut microbiome in ways that are comparable to other opioids. The research reveals that hydromorphone administration can lead to significant alterations in gut microbial composition (Abu et al., 2022; Sharma et al., 2020). The findings regarding α‐diversity are contradictory, with some studies indicating a significant decrease (Sharma et al., 2020) and others reporting no change (Abu et al., 2022). In animal models, hydromorphone treatment has been associated with shifts in the relative abundances of various bacterial taxa, including reductions in beneficial bacteria such as *Lactobacillus*, Lachnospiraceae, *Anaerostipes*, *Adlercreutzia*, *Allobaculum*, *Roseburia*, and *Oscillospira*, alongside increases in genera such as *Bacteroides*, *Enterococcus*, *Sutterella*, *Akkermansia*, *Turicibacter*, *Verrucomicrobia*, and *Clostridium* (Abu et al., 2022; Sharma et al., 2020). These microbial changes are often linked to increased systemic inflammation (Sharma et al., 2020), highlighting the need for further research to elucidate the full scope of hydromorphone's impact on gut health and its broader implications for inflammatory conditions.

### Oxycodone and methadone

Oxycodone and methadone have both been shown to significantly alter the gut microbiome, albeit in different ways. Oxycodone has been associated with a reduction in overall microbial diversity and shifts in the abundance of specific bacterial taxa (Simpson et al., 2020). For instance, the studies using rat models have demonstrated that oxycodone exposure leads to a significant decrease in both Bacteroidetes and Firmicutes at the phylum level (Simpson et al., 2020). Moreover, in female rats, oxycodone exposure has been associated with an increased relative abundance of *Butyricimonas* spp., *Bacteroidetes*, *Anaeroplasma* spp., TM7, *Enterococcus* spp., and Clostridia (Lyu et al., 2022). Conversely, male rats exhibited higher relative abundances of Coriobacteriaceae, *Roseburia* spp., *Sutterella* spp., and Clostridia following oxycodone exposure (Lyu et al., 2022). Methadone, while similar in its effects, has shown a somewhat distinct pattern of microbial changes. It is often linked to decreases in microbial diversity and an increase in the abundance of Erysipelotrichaceae, Peptostreptococcaceae, Akkermansiaceae, Lactobacillaceae, Sutterellaceae, *Eubacterium coprostanoligenes*, Anaerovoracaceae, Monoglobaceae, and Eggerthellaceae, with a concurrent reduction in *Bacteroidetes* and Actinobacteria (Grecco et al., 2021). In human studies, methadone use has been associated with an increase in Actinobacteria, particularly Bifidobacteriaceae, *Bifidobacterium bifidum*, and *Bifidobacterium longum*, while leading to a decrease in Verrucomicrobia, Akkermansiaceae, and *Akkermansia muciniphila* (Cruz‐Lebrón et al., 2021). Collectively, the impacts of oxycodone and methadone on gut microbiota underscore the complex interplay between opioid use and gut microbiota. As opioid therapies continue to evolve, understanding their differential impacts on gut microbiota will be crucial for optimizing treatment strategies and minimizing adverse effects on gut health.

### Fentanyl

Fentanyl, a potent synthetic opioid, has been increasingly studied for its effects on the gut microbiome due to its widespread use in pain management and high potential for misuse. Research indicates that fentanyl can induce significant alterations in gut microbial composition, similar to other opioids. Specifically, a rat study revealed that fentanyl administration resulted in an increased abundance of *Ruminococcus* in males and *Prevotella* in females, while concurrently causing a decrease in Verrucomicrobia and *Akkermansia* in male rats (Ren & Lotfipour, 2022). These shifts in microbial composition are thought to contribute to the systemic effects of fentanyl, including its impact on immune function and the exacerbation of opioid‐induced side effects such as constipation and increased susceptibility to infections. Understanding fentanyl's distinct influence on the gut microbiome is crucial for developing targeted therapies to mitigate its adverse effects on gut health.

Across the studies reviewed, significant shifts in the gut microbial community have been observed, characterized by the overgrowth of pathogenic bacteria and the reduction of beneficial commensal bacteria. These alterations in microbial populations due to OUD can also potentially contribute to broader physiological and pathological outcomes. For instance, opioid use has been implicated as a potential risk factor in exacerbating *Clostridiodes difficile* infection (CDI) severity (Mora et al., 2012). For example, the increased prevalence of *Enterococcus faecalis* which can augment the tolerance of morphine analgesic effects in mice (Wang et al., 2018), has shown a positive correlation with CDI burdens through enhancing *C. difficile* colonization and survival (Smith et al., 2022). However, conflicting findings have emerged suggesting that opioid exposure at predominantly low‐to‐moderate levels may not amplify CDI severity (Chowdhry et al., 2021). Additionally, over‐prescription of opioids has been implicated in increasing susceptibility to other hospital‐acquired infections, including those related to *Citrobacter rodentium* (Wang, Meng, et al., 2020a). Clinical studies have shown that OUD may escalate disease severity of patients with Crohn's disease (CD), with reports indicating a 25% increase in disease recurrence and a 15% increase in remission rates among opioid users (Chen et al., 2023). This heightened severity may be attributed to the increased presence of opportunistic pathogens such as Enterobacteriaceae and *Enterocloster* in individuals with OUD, which have been associated with IBD (Baldelli et al., 2021). Hence, the altered gut microbial composition associated with OUD may also increase the susceptibility to a range of infections and diseases.

## OUD AND METABOLIC ALTERATIONS

Recent research has underscored significant functional changes associated with OUD. The alterations in the gut microbiome can extend to metabolic disruptions affecting critical metabolites such as short‐chain fatty acids (SCFAs), produced by bacterial‐mediated digestion of dietary fibres. The most abundant short‐chain fatty acids SCFAs are acetate, propionate, and butyrate. Members of the Bacteroidetes phylum primarily produce acetate and butyrate, while the Firmicutes phylum is associated with butyrate production (Macfarlane & Macfarlane, 2003). SCFAs‐producing bacteria, including *Alistipes*, *Roseburia*, *Eubacterium rectale*, and *Faecalibacterium prausnitzii* (David et al., 2014), have been found to be reduced in individuals with OUD (Gicquelais et al., 2020; Greenberg et al., 2024). Furthermore, SCFAs play a crucial role in maintaining gut barrier integrity and modulating immune responses, yet individuals with OUD have been found to exhibit reduced levels of SCFAs (Cruz‐Lebrón et al., 2021; Yang, Xiong, et al., 2021a). Notably, these small metabolites possess the ability to cross the blood–brain barrier, impacting its integrity (Braniste et al., 2014; Mitchell et al., 2011). In a study by Hofford et al. (2021), the replacement of SCFAs reversed the behavioural and transcriptional effects of morphine depletion in mice, which identifies SCFAs as a crucial mediator in gut–brain signalling. Furthermore, SCFAs can affect circulating immune cells in the brain (Arpaia et al., 2013; Berer et al., 2011). SCFAs and lipopolysaccharides (LPS) produced by gut microbiota can indirectly activate the vagal nerve by binding to their receptive receptors, G protein coupled receptors (GPCR) and toll‐like receptor 4 (TLR4), respectively (Bonaz et al., 2018). LPS can trigger inflammatory processes and influence mood and behaviour (Bonaz et al., 2018). For example, Bhave et al. (2017) found that activation of the connexin‐purinergic pathway in enteric glia vis LPS was a significant source of cytokine release with chronic morphine treatment. Given the myriad of potential cellular mechanisms and the known effects on brain function and behaviour, the SCFAs are positioned as a target of great mechanistic interest in the gut–brain axis implicated in OUD.

Bile acids, the main metabolites of cholesterol in the liver and traditionally recognized for their role in lipid digestion and absorption, have also emerged as metabolites influenced by the gut microbiota (Larabi et al., 2023; Li et al., 2012; Ramírez‐Pérez et al., 2017). Following synthesis in the liver, primary bile acids undergo conjugation with taurine, glycine, or sulphates, and are subsequently secreted into bile and stored in the gall bladder until they are released into the small intestine. In the gut, they are deconjugated by bile salt hydrolase (BSH) activity of the gut microbiota, and further metabolized to secondary bile acids (Guzior & Quinn, 2021). Beyond their role in digestion, bile acids act as signalling molecules that modulate various physiological processes, including lipid metabolism, glucose homeostasis, and immune responses (Li et al., 2012). The gut microbiota influences the composition of bile acids, which in turn, can affect host metabolism through the activation of nuclear receptors, such as the farnesoid X receptor (FXR) and the G protein‐coupled bile acid receptor (TGR5) (Ferrell et al., 2016). These receptors regulate the expression of genes involved in lipid and glucose metabolism, as well as inflammation, thus creating a complex feedback loop between the gut microbiota, bile acids, and the host's metabolic health (Song et al., 2009). Alterations in the gut microbiota can disrupt this delicate balance, leading to dysregulation of bile acid metabolism. In individuals with OUD, this disruption is often marked by a reduction in both primary and secondary bile acids, exacerbating metabolic disturbances (Banerjee et al., 2016; Wang et al., 2018). Increased levels of secondary bile acids are essential for host resistance to infections such as CDI as they influence both sporulation and germination processes (Winston & Theriot, 2016). Thus, in individuals with OUD, the decreased levels of secondary bile acids could potentially reduce host resistance, making them more susceptible to infections such as CDI. Moreover, the composition of the gut microbiota itself is influenced by bile acids, which possess antimicrobial properties that can selectively inhibit or promote the growth of certain bacterial species (An et al., 2022). Dysregulated bile acid metabolism has also been linked with hypercholesterolemia, diabetes, and certain types of cancers (McGlone & Bloom, 2019; Tsuei et al., 2014). We hypothesize that individuals with OUD may be lacking the necessary gut microbiota (i.e., decreased BSH activity) required for deconjugation and conversion to secondary bile acids. In addition, studies investigating the metabolic consequences of opioids have revealed alterations in cholesterol metabolism, with opioids inducing the accumulation of cholesterol in the liver, as well as increased total‐cholesterol, low‐density lipoproteins (LDL), and decreased high‐density lipoproteins (HDL) levels in serum (Banerjee et al., 2016; Maccari et al., 1991; Valverde‐Filho et al., 2015). Opioids have also been considered a risk factor for the development of cardiovascular disease (CVD) (Chow et al., 2021; Khodneva et al., 2016; Krantz et al., 2021) and thus require further investigation.

In addition to SCFAs and bile acids, gut bacteria also produce tryptophan, serotonin, dopamine, and gamma‐aminobutyric acid (GABA), which play important roles in the brain as neurotransmitters or their precursors (Wall et al., 2014). Dysfunctional serotonin signalling, regulated by gut microbiota, has been implicated in GI and mood disorders (Terry & Margolis, 2017), highlighting the intricate interplay between the gut microbiome, neurotransmitter synthesis, and central nervous system (CNS) function in individuals with OUD. Thus, understanding the mechanisms underlying changes in these metabolites is essential for developing targeted interventions to mitigate metabolic and functional consequences of chronic opioid use.

Microbial transformation of opioids can also produce various metabolites that influence both drug efficacy and side effects. Opioids undergo biotransformation, resulting in the generation of secondary metabolites that can affect host physiology. Initially, opioids are subject to phase‐I metabolic transformations such as O‐dealkylation, N‐dealkylation, ketoreduction, or deacetylation, resulting in phase‐I metabolites (Lötsch, 2005). Subsequently, phase‐II metabolites are produced through glucuronidation or sulphation (Lötsch, 2005). For example, morphine primarily undergoes hepatic glucuronidation via uridine 5′‐diphospho‐glucuronosyltransferase (UGT) phase II enzymes, producing morphine‐3‐glucuronide (M3G) and morphine‐6‐glucuronide (M6G), which have been shown to modulate opioid receptor activity differently compared to their parent drugs (Andersen et al., 2003; Coates & Lazarus, 2023; Lötsch, 2005). Codeine, which is a weaker μ‐opioid receptor agonist, is metabolized into morphine (about 10%) and other metabolites such as codeine‐6‐glucuronide and norcodeine (Lötsch, 2005). Tramadol is converted into its active metabolite O‐desmethyltramadol, which activates μ‐opioid receptors more effectively than tramadol (Lötsch, 2005; Smith, 2009). These metabolites can influence the pharmacokinetics and dynamics of opioids, potentially affecting drug efficacy and the severity of side effects. Understanding these metabolites is essential for optimizing opioid therapy, managing GI disturbances, and developing strategies to reduce opioid‐related adverse effects through targeted microbiome modulation. Future research is needed to uncover how these metabolites impact the gut microbiome, its metabolites, and gut integrity.

## OUD AND IMPAIRED GUT INTEGRITY

The gut mucus, an essential barrier between, and nutrient source for, the host and the diverse microorganisms residing in the gut, is crucial in health and disease management (Cornick et al., 2015; König et al., 2016). The alterations in gut microbiome composition and functions induced by opioid use can also lead to changes in gut‐barrier permeability, and bacterial translocation, with potential implications for systemic inflammation (Acharya et al., 2017; Banerjee et al., 2016; Meng et al., 2013; Yang, Xiong, et al., 2021a). For example, increased levels of *Enterococcus* in individuals with OUD can contribute to intestinal inflammation and compromise the epithelial barrier by disrupting tight junction function (Steck et al., 2011). In contrast, *Lactobacillus* spp., often reduced by OUD exposure, can enhance the intestinal barrier and reinforce tight junction integrity (Blackwood et al., 2017). Metabolites such as SCFAs, also play a critical role in maintaining mucosal epithelial and immune homeostasis (Willemsen et al., 2003); therefore, their reduction in opioid use can contribute to gut permeability (“leaky gut”) and systemic inflammation. A study by Cruz‐Lebrón et al. (2021) has shown the therapeutic potential of SCFAs produced by *Akkermansia muciniphila* in intestinal tissue integrity, as they regulate tight junction protein expression. Heroine, for example, has been shown to decrease tight junction proteins leading to impaired gut barrier integrity (Yang, Xiong, et al., 2021a). Recent reports also indicate that bile acid metabolism has a significant role in the manifestation of gut barrier pathology and resultant inflammation (Shi et al., 2023; Sun et al., 2021). The disruption of the gut barrier and the translocation of bacteria have also been implicated in systemic inflammation. For example, Meng et al. (2013) reported that the disruption of the gut epithelial barrier as a result of morphine‐mediated activation of TLR‐4 and TLR‐2 on the epithelial cells can lead to bacterial translocation. Opioids have also been shown to increase apoptosis and elevate inflammatory cytokines (Ghosh et al., 2023). In a study by Meng et al. (2015), IL‐17A neutralization protected barrier integrity and improved survival in morphine‐treated animals. Therefore, disruptions in the gut microbiome can exacerbate gut integrity issues by disrupting the balance of beneficial bacteria, weakening the intestinal barrier, and increasing susceptibility to inflammation and permeability. Expanding research scopes to include the gut mucus layer is vital for a more comprehensive understanding of OUD's effect on the gut and overall health.

## GUT–BRAIN AXIS IN OUD

**FIGURE 2:**
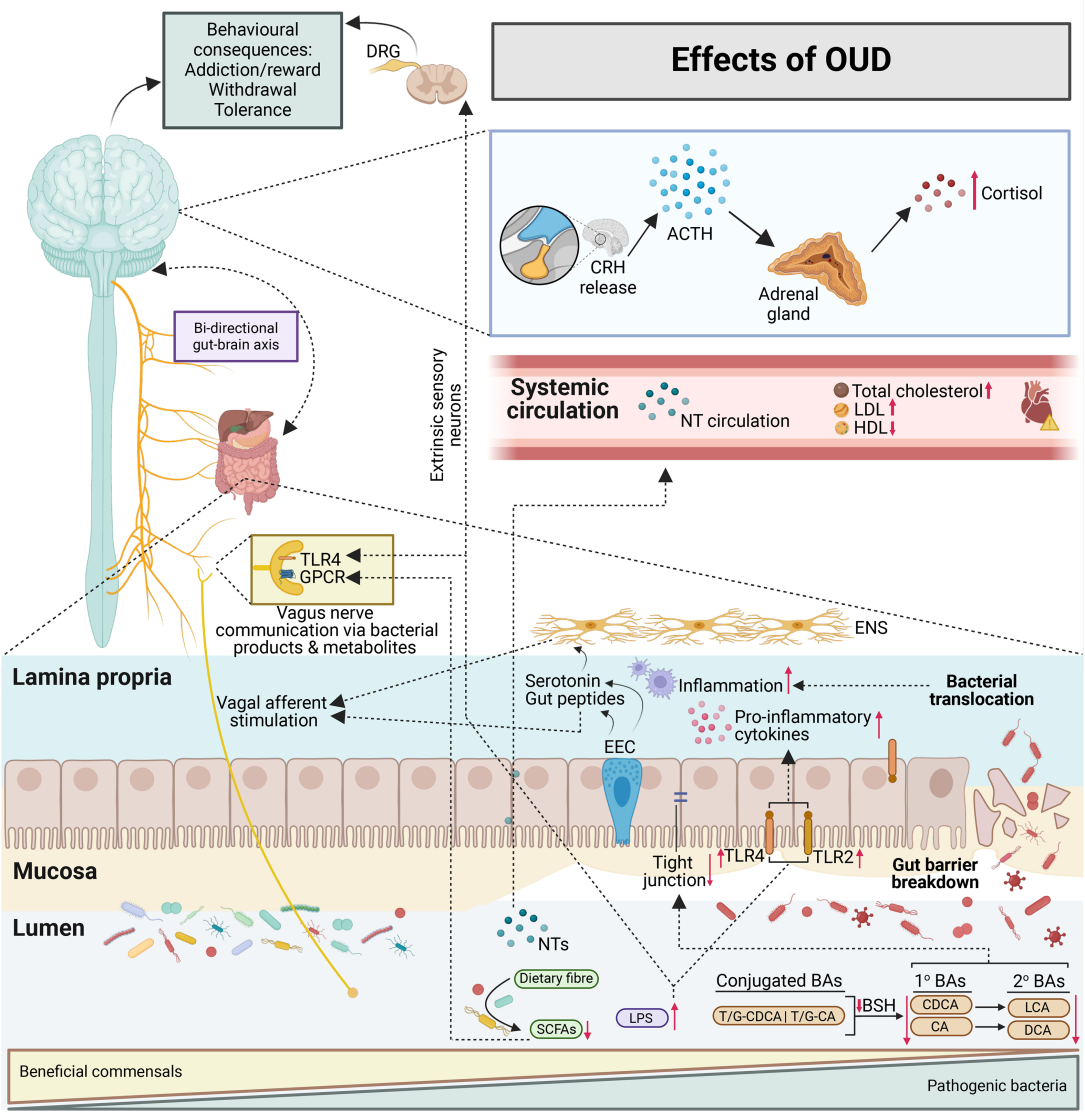
The multifaceted systems collectively influence the overall state and function of the gut and brain and their implications in opioid use disorder (OUD). Potential mechanisms by which OUD can alter the gut ecosystem (i.e., the gut microbiota, metabolites, mucus), the brain via the gut–brain axis, and overall systemic health. LDL, low‐density lipoprotein cholesterol; HDL, high‐density lipoprotein cholesterol; BAs, bile acids; 1° BAs, primary bile acids; 2° BAs, secondary bile acids; CDCA, chenodeoxycholic acid; CA, cholic acid; LCA, lithocholic acid; DCA, deoxycholic acid; T/G, taurine/glycine; BSH, bile salt hydrolase; SCFAs, short chain fatty acids; LPS, lipopolysaccharides; NTs, neurotransmitters; TLR2 and 4, toll‐like receptors 2 and 4; GPCR, G protein coupled receptor; DRG, dorsal root ganglia; EEC, enteroendocrine cell; ENS, enteric nervous system; CRH, Corticotropin‐releasing hormone; ACTH, adrenocorticotropic hormone. Image created with BiorRender.com.

The gut environment constitutes a dynamic and intricate ecosystem shaped by various interacting elements including the gut microbiota, metabolites, and mucus, all of which bidirectionally communicate with the brain via the gut–brain axis. These elements collectively influence the overall state and function of the gut and play significant roles in OUD, as illustrated in Figure 2.

### From the brain to the gut

Excitingly, emerging research has revealed the interconnectedness of the gut microbiota, the brain, and various aspects of the gut–brain axis (Chang et al., 2022; Mayer et al., 2014), which play pivotal roles in mediating physiological responses, and have implications for SUDs, including OUD (Rueda‐Ruzafa et al., 2020; Simpson et al., 2022). Traditionally, the central effects of opioids, such as analgesic tolerance and hyperalgesia, were believed to be exclusive to the CNS; however, recent findings suggest bidirectional communication between the gut and the brain in opioid addiction (Galligan & Sternini, 2017; Rueda‐Ruzafa et al., 2020). Through direct connections via the vagus nerve and indirect signalling pathways mediated by neurotransmitters and neuropeptides, the brain influences GI functions such as motility, intestinal transit and secretion, gut permeability, and immune response (Martin et al., 2018). Additionally, microglia, the resident macrophages of the CNS, become significantly more activated during opioid exposure (Antoine et al., 2022; Green et al., 2022), which have been shown to play a role in morphine tolerance through the release of pro‐inflammatory cytokines including interleukin (IL)‐1β and tumour necrosis factor (TNF)‐α (Yang, Sun, et al., 2021b). Moreover, stress and mental health disorders such as depression and anxiety are also intricately linked to SUDs including OUD and have been increasingly recognized for their significant impact on the gut microbiome. Research indicates that stressors, depression, and anxiety can lead to altered gut microbiota composition and function, which can in turn contribute to GI symptoms and exacerbate the severity of mental health symptoms (Kumar et al., 2023). Thus, the dynamic interplay between the brain and the gut, modulated by diverse signalling pathways and exacerbated by factors like stress and mental health disorders, underscores the complexity of the gut–brain axis in OUD, offering promising avenues for further exploration and therapeutic interventions.

### From the gut to the brain

The gut microbiome, intricately linked to the brain via neural, endocrine, immune, and metabolic pathways, plays a crucial role in mediating the effects of OUD on brain function and behaviour (Cryan & Dinan, 2012; Simpson et al., 2022). It plays a role in opioid reward and sensory responses, suggesting a bidirectional relationship between opioid use and the gut microbiome (Blakeley‐Ruiz et al., 2022; Lee et al., 2018). Thus, the changes in the gut microbiome may not only be a consequence of OUD but may play a role in mediating behavioural responses to opioids (Meckel & Kiraly, 2019). For example, inflammation and metabolic disturbances may influence the brain's reward pathways, making it more difficult for individuals to achieve and maintain abstinence. In a study by Hofford et al. (2021), it was shown that alterations in microbiome composition and metabolites drive behavioural and transcriptional responses to morphine. Additionally, in chronic morphine‐treated mice that received antibiotics, dorsal root ganglia (DRG) neurons, which serve as primary relay stations between the periphery and the brain, exhibited reduced neuronal excitability, suggesting a lack of tolerance development in these nociceptors (Kang et al., 2017). These findings indicate that alterations in the gut microbiome can influence opioid tolerance in the cell bodies of extrinsic sensory neurons. Opioid tolerance to analgesia has also been shown to be transferrable via faecal transfers from an opioid‐dependent mouse model into opioid‐naïve mice, implicating the gut microbiota in opioid tolerance development (Kang et al., 2017; Lee et al., 2018). Enteric glia, key players in mediating GI functions and immune response, have been found to exhibit enhanced purinergic P2X receptor activity in response to long‐term morphine treatment in mice (Bhave et al., 2017), contributing to gut–brain axis dysregulation in opioid addiction. In addition, enteroendocrine cells (EECs) in the epithelium, which consist of 1% of the intestinal epithelial cells, can help relay luminal signals to the vagal nerve (Bonaz et al., 2018). For example, EECs can release gut peptides such as glucagon‐like peptide‐1 (GLP‐1), which can activate vagal neurons (Gribble & Reimann, 2016). The EECs also express receptors for bacterial products and can release serotonin and activate 5‐HT3 receptors on vagal afferent fibres, thus affecting gut–brain communication (Li et al., 2000). Additionally, the hypothalamic–pituitary–adrenal (HPA) axis involved in stress response, is influenced by the gut microbiome, further implicating its role in the gut–brain axis dysregulation observed in OUD (Bock, 2020; Cryan & Dinan, 2012). The HPA axis begins with the activation of the hypothalamus, releasing corticotropin‐releasing hormone (CRH) and inducing the anterior pituitary gland to release adrenocorticotropic hormone (ACTH), which stimulates the adrenal cortex to produce Glucocorticoids (GCs) (Sheng et al., 2021). GCs such as cortisol bind to GC receptors present on many cells throughout the body, including microglial cells, resulting in various homeostatic functions (Timmermans et al., 2019), as well as playing a critical role in the development of addiction. Thus, the gut–brain axis plays a crucial role in the development, progression, and severity of OUD. Understanding the interplay between the gut microbiome and the brain is essential to mitigate the neurobiological and behavioural consequences of opioid addiction.

## CHALLENGES AND OPPORTUNITIES

This review underscores the multifaceted nature of the gut environment implicated in OUD, compounded by the complexity of the gut–brain axis. Future studies should aim to comprehensively unravel the underlying mechanisms driving the bidirectional interactions between OUDs and the gut ecosystem (i.e., microbial composition, metabolites, and gut barrier integrity), alongside elucidating their intricate relationship with the gut–brain axis. Specifically, further investigation is warranted to understand how the gut ecosystem and its alterations can lead to the development of analgesic tolerance and addiction such as OUD. Additionally, it is imperative to evaluate the impact of OUD on the gut, as well as any systemic implications affecting overall health and susceptibility to diseases. For example, OUD has been identified as a potential causal role in the risk for depression and anxiety disorders (Rosoff et al., 2021). Consequently, the resulting dysbiosis of the gut microbiome due to OUD may be implicated in the pathophysiology of stress‐related disorders. Therefore, understanding the interplay between stress, mental health, and the gut microbiome is crucial for developing novel therapeutic strategies that target both the gut and the brain to promote overall well‐being and mental health resilience.

Research studies on OUD encounter numerous challenges that impede our understanding and management of this pervasive public health issue. One significant hurdle is the multifaceted nature of OUD, encompassing a diverse array of risk factors, comorbidities, and treatment responses. For example, OUD prevalence can vary across different demographic groups. Age can play a significant role, with younger individuals often being at higher risk due to factors such as peer pressure, experimentation, and susceptibility to addiction (Shanahan et al., 2021). Additionally, sex differences exist, with males historically showing higher rates of opioid misuse and dependence; however, recent trends suggest a narrowing in this gap as gonadal hormones may be driving behavioural sex differences in OUD (Bagley et al., 2020; Knouse & Briand, 2021; McHugh et al., 2013). Moreover, ethnicity and socio‐cultural factors can impact opioid use patterns, with certain ethnic groups experiencing disparities in access to healthcare, socioeconomic status, and exposure to trauma, all of which can contribute to differential rates of OUD within populations (Essien‐Aleksi et al., 2023).

Diet (David et al., 2014; Singh et al., 2017) and demographic factors such as age (Claesson et al., 2012; Kim & Jazwinski, 2018; van Tongeren et al., 2005), sex (Chen et al., 2016; Takagi et al., 2019), and ethnicity (Biswas et al., 2013) can also affect the gut microbiome and its function, further complicating the relationship between OUD and the gut ecosystem. Emerging evidence suggests that diet plays a crucial role in shaping the composition and function of the gut microbiome, which in turn can influence various aspects of health and disease, including OUD. Different dietary patterns, such as the Mediterranean, high‐fibre, and Western diet, can lead to variations in gut microbial diversity and composition, influencing metabolic health, gut integrity, immune response, and susceptibility to diseases (David et al., 2014; Krznarić et al., 2019; Zhang, 2022). Moreover, dietary patterns can significantly impact insulin sensitivity by modulating the gut microbiome and its metabolic functions, further influencing the risk of developing metabolic disorders (Martins & Conde, 2022). For instance, a Western diet, characterized by high levels of fats and sugars, is linked to an increase in Bacteroidetes and a decrease in Firmicutes and *Bilophila wadsworthia* (sulphite‐reducing microorganisms) (David et al., 2014). This dietary pattern is associated with dyslipidaemia and heightened inflammation, which may exacerbate symptoms and complications of OUD (Devkota et al., 2012; Qin et al., 2012). In contrast, the Mediterranean diet has been demonstrated to boost SCFAs, improve obesity, reduce inflammation, and address related metabolic disturbances (Garcia‐Mantrana et al., 2018; Krznarić et al., 2019). Individuals with OUD often exhibit a strong preference for sweet, convenient foods, and decreased intake of vegetables, fruits, and grains (Booth et al., 2006; Wiss, 2019). This dietary imbalance leads to significant nutritional deficiencies and poor nutritional status, characterized by low levels of micronutrients such as iron, selenium, and potassium, coupled with elevated sodium intake (Ii et al., 2016; Kolarzyk et al., 2005). Conversely, periods of abstinence are frequently accompanied by binge eating behaviours (Canan et al., 2017). In addition, the studies have shown both low and high energy intake in individuals with OUD, underscoring the need for adequate energy and micronutrient intake to prevent comorbidities like cardiovascular diseases, respiratory issues, diabetes, and cancer (Kolarzyk et al., 2005; Waddington et al., 2015). In the context of OUD, interventions such as probiotics, prebiotics, supplementation, and dietary modifications may potentially reduce cravings, alleviate withdrawal symptoms, and support recovery by promoting a healthier gut microbiome (Ait‐Belgnaoui et al., 2012; Liu et al., 2016; Molavi et al., 2022; Zhang et al., 2019). Although opioid‐induced changes in the gut microbiome can be profound, there is potential for partial recovery. For instance, a study by Zhang et al. (2020) demonstrated that probiotics alleviated morphine tolerance in mice and partially restored partial gut microbial components (Zhang et al., 2019). Similarly, dietary polyphenols have been shown to reduce morphine tolerance (Han et al., 2014; Osman et al., 2023), induce goblet cell differentiation, increase mucus layer thickness, and decrease inflammation (Rodríguez‐Daza & de Vos, 2022; Wang, Li, et al., 2020b), indicating their potential for further exploration in the prevention and treatment of OUD. Additionally, an n‐3‐PUFA‐enriched diet has been found to ameliorate oxycodone‐seeking behaviours, enhance gut microbial diversity, lower the basal activation state of microglia, and reduce anxiety‐related opioid‐seeking behaviour and relapse (Hakimian et al., 2019). Aging also affects the gut microbial community and is associated with increased gut dysbiosis due to the accumulation of disorders, changes in diet, a decrease in exercise and mobility, and the use of certain medications (Claesson et al., 2012; Kim & Jazwinski, 2018). Aging can also affect the immune system, with systemic inflammation being one of the hallmarks of aging (Chmielewski, 2018). Considering these dynamic interactions among diet, age, sex, and ethnicity—and their collective impact on the gut microbiome—offers promising insights for developing effective preventative and therapeutic strategies for OUD.

The clandestine nature of opioid misuse can often lead to underreporting or concealment of drug use, complicating efforts to obtain reliable data on opioid consumption patterns and treatment needs. Furthermore, ethical considerations surrounding participant confidentiality and privacy present challenges in recruiting and retaining individuals with OUD in research studies (Clapp et al., 2023). Stigma and discrimination against individuals with OUD also pose barriers to engagement in research and access to treatment, exacerbating disparities in care and hindering efforts to address the opioid crisis effectively (Adams et al., 2021). Overcoming these challenges requires a collaborative interdisciplinary approach, innovative research methodologies, and community engagement strategies to foster trust and collaboration between researchers, healthcare providers, policymakers, and individuals affected by OUD.

While addressing the opioid epidemic at its source and advocating for physicians to utilize prescription‐drug monitoring programs to mitigate opioid abuse and overdoses, alongside promoting non‐opioid pain management alternatives, understanding the mechanisms driving the bidirectional interactions between OUDs and the gut ecosystem holds promising avenues for the prevention and treatment of OUDs. Although gut microbiota are susceptible to alterations induced by opioid use, their responsiveness to various manipulative approaches underscores their potential as promising candidates for prevention and treatment strategies. Next‐generation sequencing (NGS) technology and “omics” data such as human genomic, metabolomics, glycomics, transcriptomics, and proteomic data have revolutionized the field of microbiome; however, many limitations still exist. For example, OUD studies often utilize 16S rRNA sequencing which can lead to a uni‐kingdom outlook on bacteria; however, it is important to consider all aspects of life including fungi, protozoa, and viruses in the gut. Despite their importance, these microorganisms are often frequently overlooked in OUD‐related research. Moreover, existing studies have not systematically compared the effects of different opioids on microbial diversity and abundance, underscoring the need for future research to directly assess and compare these impacts across various opioids to enhance our understanding of their differential effects on the gut microbiome. Therefore, future studies should aim to adopt broader metagenomic approaches that can widen the scientific lens into a multi‐kingdom view, thus providing a more comprehensive understanding of the interplay between OUD and the gut microbiome. Nevertheless, metagenomic approaches also contain limitations that warrant careful consideration. For example, a significant proportion of the data cannot be assigned a function due to a lack of close matches in reference databases, specifically viral data (Liu et al., 2022).

Future research should focus on several key areas to advance our understanding of the interactions between OUD and the gut microbiome. Crucial areas for further exploration include the use of animal models, gut‐on‐a‐chip and organoid studies to dissect the specific pathways and interactions at play. These innovative approaches can simulate the human gut environment, aid in confirming causality and allow for a deeper investigation into the bidirectional relationship between OUD and the gut ecosystem. Research utilizing brain organoids has also emerged as a valuable tool to study the neurological effects of OUD. For example, brain organoids have been used to investigate opioid‐induced neurotoxicity, cell‐type‐specific responses, alterations in neural circuitry, and the impact of chronic opioid exposure on neural development (Dwivedi & Haddad, 2024; Kim et al., 2024; Li et al., 2024). Such studies offer critical insights into the pathophysiological changes in the brain associated with OUD, helping to elucidate the mechanisms underlying opioid addiction and its long‐term effects on brain function. In addition, opportunities for future research include the development of targeted interventions aimed at restoring gut microbial balance and their metabolites (e.g., SCFAs, bile acids, and neurotransmitters). In addition, although gut mucus glycosylation alterations have been associated with conditions such as colitis, colonic cancer, and IBD (Barrios et al., 2016; Gudelj et al., 2018; Lauc et al., 2016; Rhodes, 1996), this topic has received virtually no experimental validation when assessing the relationship between OUD and the gut microbiome. Additionally, recent attention has been directed towards faecal microbiota transplantation (FMT) as a possible treatment for early recovery (Zhang & Roy, 2021), with promising results demonstrated in a phase 1 clinical trial for alcohol use disorder (Bajaj et al., 2021). However, effective implementation faces challenges, such as variability in individual microbiome compositions, potential adverse effects, and questions of long‐term efficacy. To maximize the benefits of such microbiome‐based therapeutics, it is crucial to address technical commercialization bottlenecks, ensure affordability, and establish a comprehensive framework for sustainable microbiome medicine (Zhang et al., 2023). Moreover, the development of preventive strategies to mitigate OUD and its side effects represents an important area for investigation. This may involve identifying biomarkers or microbial signatures predictive of susceptibility to opioid‐induced gastrointestinal dysfunction, as well as implementing early interventions to preserve gut health in at‐risk populations. For example, the potential role of nanotechnologies in screening vulnerable individuals to opioid addiction, as well as promise in therapeutic approaches has been highlighted (Ashkarran et al., 2020; Mahmoudi et al., 2018). In a study by Ashkarran et al. (2020), the optical images of levitating human plasma proteins via magnetic levitation (Maglev) were used to detect OUD. Taken together, such strategies could alleviate the burden on healthcare systems by decreasing the incidence of substance‐related health issues such as OUDs and enhancing the efficacy of treatment modalities.

## AUTHOR CONTRIBUTIONS

**Negin Kazemian:** Conceptualization; writing – original draft; writing – review and editing; visualization. **Sepideh Pakpour:** Conceptualization; supervision; writing – review and editing.

## FUNDING INFORMATION

This research received no external funding.

## CONFLICT OF INTEREST STATEMENT

The authors declare no conflicts of interest.
